# Macular Pigment Optical Density as a Measurable Modifiable Clinical Biomarker

**DOI:** 10.3390/nu16193273

**Published:** 2024-09-27

**Authors:** Abdul Masri, Mohammed Armanazi, Keiko Inouye, Dennis L. Geierhart, Pinakin Gunvant Davey, Balamurali Vasudevan

**Affiliations:** 1 Arizona College of Osteopathic Medicine, Midwestern University, Glendale, AZ 85308, USA; abdulaziz.masri@midwestern.edu; 2 College of Medicine, University of Saskatchewan, Saskatoon, SK S7N 5A2, Canada; m.armanazi@usask.ca; 3 College of Optometry, Western University of Health Sciences, Pomona, CA 91766, USA; keiko.inouye@westernu.edu; 4 EyePromise, LLC, Chesterfield, MO 63005, USA; dgierhart@zeavision.com; 5 Arizona College of Optometry, Midwestern University, Glendale, AZ 85308, USA; bvasud@midwestern.edu

**Keywords:** MPOD, lutein, zeaxanthin, biomarker, glaucoma, diabetic retinopathy

## Abstract

Background: Carotenoids are present throughout retina and body its dense deposition leads to an identifiable yellow spot in the macula. Macular pigment optical density (MPOD) measured in the macula is vital to macular well-being and high-resolution visual acuity. MPOD has also been associated with various health and disease states. We sought to review the literature on this topic and summarize MPODs role as a measurable modifiable clinical biomarker, particularly as a measure of the eye’s antioxidant capacity in the context of oxidative damage and retinal ischemia. Methods: A literature review collated the articles relevant to MPOD, carotenoid intake or supplementation, and their influence on various health and disease states. Results: Literature reveals that MPOD can serve as a reliable biomarker for assessing the retinal defense mechanisms against oxidative stress and the deleterious effects of excessive light exposure. Elevated MPOD levels offer robust protection against the onset and progression of age-related macular degeneration (AMD), a prevalent cause of vision impairment among the elderly population. MPOD’s implications in diverse ocular conditions, including diabetic retinopathy and glaucoma, have been explored, underscoring the real need for clinical measurement of MPOD. The integration of MPOD measurement into routine eye examinations presents an unparalleled opportunity for early disease detection, precise treatment planning, and longitudinal disease monitoring. Conclusions: Longitudinal investigations underscore the significance of MPOD in the context of age-related ocular diseases. These studies show promise and elucidate the dynamic nuances of MPOD’s status and importance as a measurable, modifiable clinical biomarker.

## 1. Introduction

### 1.1. Structure

The macular pigment, a yellowish deposit in the central retina, arises from the collective presence of carotenoid pigments strategically accumulated within the macular region [1]. These pigments exhibit distinct absorption spectra, aiding their identification and quantification [2]. Lutein, zeaxanthin, and meso-zeaxanthin together comprise the macular pigment and are present throughout the retina, although centrally, its deposition is substantially higher and thus the visibility of a central yearly spot in humans [2,3]. Lutein, a xanthophyll carotenoid, is known for its antioxidant abilities and light-filtering properties. Zeaxanthin, a close relative, exhibits similar characteristics and is particularly enriched in the central foveal region of the macula [4]. Meso-zeaxanthin, although structurally akin to zeaxanthin, is found in extremely lower quantities, if at all, from dietary sources but is synthesized from lutein in the retina [1]. This interconversion of lutein to meso-zeaxanthin may be especially advantageous for diets that are dominated by lutein-rich sources [1]. Notably, however, this conversion is not shown in other locations, and in particular, meso-zeaxanthin is starkly absent in the brain [5]. Collectively, these pigments bolster ocular defense mechanisms by quenching singlet oxygen and reactive oxygen species and absorbing blue light, thereby protecting against oxidative stress and potential retinal damage [6].

### 1.2. Nutrition

The journey of these pigments into the retina begins with dietary intake. Rich sources of lutein and zeaxanthin (L/Z) include leafy green vegetables such as spinach, kale, and collard greens, along with other vibrant fruits and vegetables [3]. However, meso-zeaxanthin’s primary origin lies within the retina, where it is synthesized from lutein, as it is not commonly found in substantial quantities within typical diets [1]. The intricate interplay between dietary intake, transport, and metabolism influences the availability of these carotenoids for ocular uptake. Bioavailability studies reveal that factors such as food matrix, cooking methods, and individual genetics can affect the extent to which these carotenoids are absorbed and utilized by the body [7,8,9,10].

To better understand the effects of L/Z intake on macular pigment density, it is important to know about the dietary sources of L/Z. In the past, nutritional analysis has reported L/Z as one value as analytical procedures had not permitted for the evaluation of them separately. Perry et al. have conducted testing to determine their individual quantities in food [11]. Given that L/Z accumulates in different regions of the retina and that they serve different functions, it is important to assess their individual quantity [4,12,13]. As seen in Table 1, there is a stark difference in the foods that contain L/Z, with most foods containing lutein but not zeaxanthin. This in part explains why dietary intake in the standard American diet is lower in zeaxanthin and higher in lutein, despite there being a higher level of zeaxanthin in the central fovea, thus emphasizing its importance in health maintenance of the eye.

The bioavailability of carotenoids has been shown to be significantly increased when consumed with foods containing fat [7,8]. Despite eggs having a lower L/Z content, their fat content allows them to significantly increase L/Z levels. A study by Goodrow et al. showed that consuming one egg per day over five weeks increased plasma levels of lutein by 26% and zeaxanthin by 38% [14]. It has also been shown that the bioavailability of carotenoids decreases due to competition for absorption when different carotenoids are consumed in the same meal [10,15]. Heat plays an interesting role as it has been shown to decrease carotenoid content but significantly increases carotenoid bioavailability [8]. Dietary fiber has been shown to have a negative effect on the absorption of carotenoids, as seen in Riedl et al., with a 40–74% decrease in plasma levels of lutein when these carotenoids were consumed with water-soluble fibers such as pectin, guar, and alginate [10].

## 2. In Vivo Measurement of Carotenoid Status in the Eye

### 2.1. Macular Pigment Optical Density (MPOD)

The carotenoids lutein, zeaxanthin, and meso-zeaxanthin form the macular pigment. Macular pigment optical density (MPOD) is an assessment of the strength of the presence of these carotenoids in an individual. This metric can be measured clinically, and it can be used as a clinical biomarker for ocular disease, ocular performance, and effects of systemic disease. If the macular pigment were to be used as a clinical biomarker, it would be imperative to be able to obtain accurate and consistent measurements of a patient’s MPOD.

### 2.2. Measurement of MPOD

There are several techniques to measure MPOD levels. These techniques can be split into psychophysical vs. objective techniques, each with their pros and cons. Psychophysical techniques include methods like heterochromatic flicker photometry (HFP) and minimum notion photometry (MNP). These techniques gauge macular pigment density by exploiting visual perception phenomena in response to specific stimulus conditions. While psychophysical methods offer insights into pigment, these methods do not provide information on pigment distribution. They provide pigment density estimates in the specific circular region of interest (approximately 1 degree), and they may be affected by individual variations in visual perception. Objective techniques include methods such as Fundus Reflectometry (FR), Fundus Autofluorescence (FAF), and Resonance Raman Spectroscopy (RRS). 

Heterochromatic flicker photometry (HFP) is the most widely used psychophysical method that relies on color sensitivity modulation. It involves presenting a flickering stimulus comprising two lights with different wavelengths. By varying the intensity of one light, the point at which the flicker disappears is indicative of the macular pigment’s absorption [16,17]. Its advantages include directly measuring overall macular pigment density, non-invasiveness, and relative simplicity to perform, as demonstrated by a large body of research [17]. While these psychophysical methods estimate the overall density, obtaining spatial distribution through psychophysics is time-consuming and not a clinically viable technique. Given that this technique requires patient response, it is prone to variations in individuals’ perception of flicker. However, this should not influence the key purpose of performing the test, which is to assess change over time so long as the patient performs the test as instructed. 

Fundus Reflectometry (FR) is an objective technique that measures the amount of light reflected from the fundus. Light that is reflected from a part of the retina is compared to light reflected from the fovea. Because the fovea (due to its high concentration of macular pigment) exhibits differential wavelength absorption compared to the other regions in the retina, the difference in reflected light can be used to determine the MPOD [18,19]. This technique utilizes controlled light beams and internal spectrometers to quantify lutein and zeaxanthin optical densities, providing reliable measurements for personalized supplementation strategies. Studies have compared the widely used technique of HFP to FR and found that there was a significant correlation between these techniques, indicating FR is an objective, accurate, and reliable measurement tool for MPOD [12,13,20]. 

Resonance Raman Spectroscopy (RRS) is a technique that leverages the resonance Raman scattering properties of the macular pigments to assess their concentrations [21,22]. Laser light of specific wavelengths is directed at the retina, causing the pigments to resonate and emit scattered light with altered frequencies [23]. By measuring this altered light, the density of pigments can be quantified [23]. Resonance Raman Spectroscopy offers higher specificity for detecting macular pigments such as lutein and zeaxanthin. This specificity is due to the unique vibrational signatures of these molecules, which can be detected even at low concentrations. Additionally, it does not offer the spatial distribution of macular pigment, and due to its complexity and dependence on sensitive, specialized, and expensive equipment, it can pose challenges. Furthermore, this technology is only available in certain research laboratories and is not available for clinical use. 

Fundus Autofluorescence (FAF) capitalizes on the phenomenon of autofluorescence exhibited by macular pigments. Pigments, when exposed to specific wavelengths of light, emit light of a longer wavelength [24]. FAF captures this emitted light and uses it as a surrogate marker for macular pigment density [24]. This technique is non-invasive and can be integrated into routine clinical examinations. However, the accuracy of the measurement is limited by variations in autofluorescence across individuals and by factors like aging and retinal health [19]. Davey et al. examined the precision and inter-eye-correlation of MPOD, finding that measurements using HFP had excellent short-term repeatability, and that the MPOD value of one eye could predict the value of the other eye with 89% accuracy [25]. MPOD, however, was not correlated with ocular dominance [25].

Numerous studies have used Macular Pigment Reflectometer (MPR) research prototypes to measure the retina’s carotenoid status directly [12,13,18,26,27,28,29]. The significant advantage of MPR is that it employs a technique that can successfully measure and separate the lutein and zeaxanthin optical density in vivo in a given region [12,13,18,26,27,28,29]. It uses a quartz halogen source, a series of filters (to prevent UV damage), and a Badal system to project a controlled full-spectrum (400 to 800 nm) light spot on the target tissue, which, in the case of in vivo eye measurements, is the retina. The illumination system determines the shape of the beam, which is also the fixation target for the participant, and the retinal stop determines the field of illumination, which is central one degree (i.e., approximately a 300 µm diameter). This measurement area coincides nicely with the area of measurement of HFP [12,13]; thus, one can expect a good correlation between the two. If a peripheral measurement is desired, one could give the participant a peripheral target for fixation, which would move the illumination system to various eccentricities on the retina [12]. The light reflected from the retina is collected and analyzed by a high-resolution spectrometer, and a mathematical function is used to estimate different parameters of interest. Lens optical density is directly measured and is included in the algorithm [12,13,18,26,27,28,29]. The lens model used in the current implementation of the MPR is the van de Kraats model, which returns two components for lens absorption: Kyoung and Kold. The young component is a “base” measurement, while the old represents the ongoing age-sensitive component of the lens. MPR automatically analyzes the retina’s signal using a spectrometer and produces measures of MPOD, lutein, and zeaxanthin optical density. The spectrometer must be of sufficient resolution to detect 5–6 nm shifts in the spectrum as lutein and zeaxanthin have similar-shaped curves; however, the zeaxanthin curve is shifted 10 nm towards the red spectrum. The zeaxanthin MPOD measurement includes the isomer of zeaxanthin meso-zeaxanthin, and the MPR in its current form cannot distinguish between the two. The MPR measurements can be performed as long as desired, but the device produces valid data after 10 s of bleaching time, and a total of 30 s of measurement is sufficient to produce repeatable data that correlates well with HFP without the need for pupillary dilation.

### 2.3. Systematic Measurement of Carotenoids—Weakly Associated with MPOD

The Veggie Meter uses reflection spectroscopy to assess the carotenoid levels in the skin. When light is shone onto the skin, carotenoids present in the skin absorb specific wavelengths of light, and the device measures the reflected light to quantify carotenoid levels. The examiner places their fingertip into the device, and the Veggie Meter shines a light on the skin. The device then measures the reflected light and calculates the skin carotenoid score. The Veggie Meter is primarily designed to measure skin carotenoids, which are linked to dietary intake of carotenoids [30,31]. However, its direct use for MPOD assessment is limited because it does not measure macular pigment directly. Skin carotenoids represent a composite score of carotenoids deposited in the skin via systemic circulation and are primarily rich in lycopene, not lutein or zeaxanthin [32]. Further, as expected, a recent study [33] has shown skin carotenoid measurements do not correlate with MPOD. There is a 0.64% correlation between the two (r = 0.08) [33].

High-Performance Liquid Chromatography (HPLC) serum carotenoids are an analytical technique used to separate, identify, and quantify each component in a mixture. They are highly effective for measuring serum carotenoids due to their precision and sensitivity [34]. This is considered the laboratory “gold standard” in carotenoid measurements. HPLC measures the levels of carotenoids in the blood serum, which are reflective of dietary intake and absorption. Higher levels of serum lutein and zeaxanthin are generally correlated with higher macular pigment optical density (MPOD). Thus, by measuring these carotenoids in the serum, one can infer potential MPOD levels. It should be noted that this technique can also be used for other biological tissues, but these are only of research interest and are almost never performed in clinics. The evaluation of serum carotenoid levels is always influenced by recent dietary intake. When using supplementation, it is easier and quicker to obtain increased serum levels, but it takes much longer to increase MPOD levels [35]. See Table 2 for a summary.

## 3. MPOD in Ocular and Systemic Disease

### 3.1. MPOD in Ocular Disease

**Figure 1:**
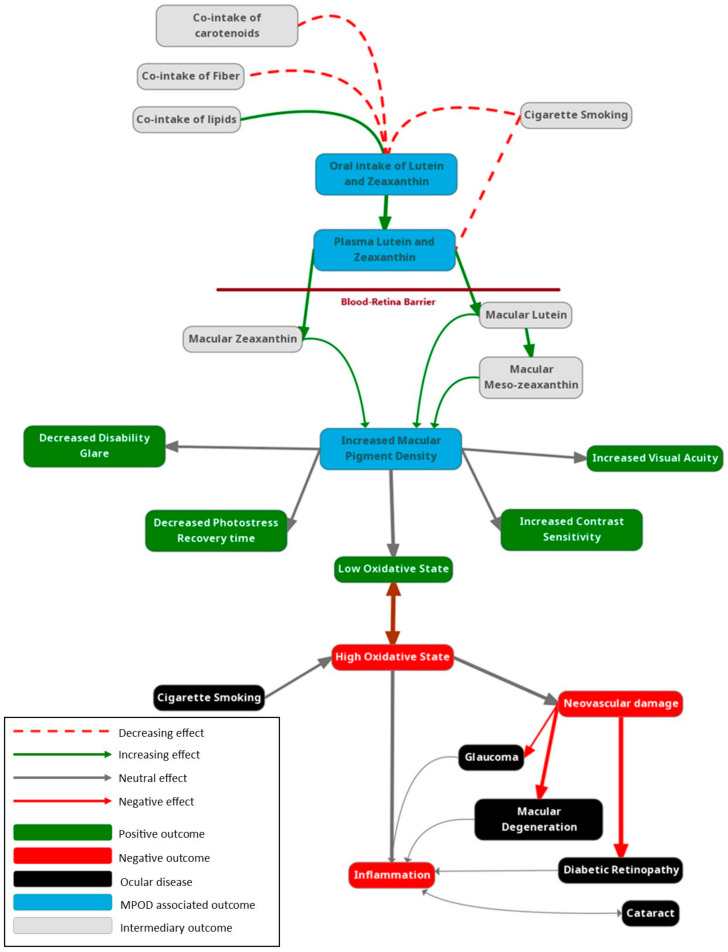
MPOD model of inflammation. This figure illustrates the hypothesized links between neovascular mechanisms in the eye and the onset of glaucoma. The dotted lines represent theoretical pathways, suggesting potential interactions between increased neovascular activity and intraocular pressure changes leading to glaucomatous damage.

Understanding the intricate relationship between MPOD and various aspects of eye health is paramount for deciphering the potential impact of pigment density on ocular diseases and conditions. This section delves into the multifaceted connection between MPOD and ocular health, exploring its protective role against AMD, its potential as a biomarker for AMD risk assessment and progression, and its influence on other ocular conditions such as diabetic retinopathy and glaucoma. Figure 1 illustrates how environmental and disease processes interact to increase or decrease MPOD, demonstrating how MPOD can be used as a clinical biomarker for many ocular and systemic conditions.

### 3.2. MPOD and Age-Related Macular Degeneration

Age-related macular degeneration (AMD), a leading cause of irreversible vision loss in older adults, underscores the importance of exploring potential protective factors [36]. Early signs of AMD are present in a quarter of the population older than 65, increasing the risk of developing late AMD [37]. Late AMD is the stage of AMD that affects vision, and 7% of individuals over 75 years old will develop late AMD over the next 10 years of their life [37]. Modern medical interventions include primarily anti-VEGF and rarely photodynamic therapy. These medical treatments present limitations in delaying and reversing the retinal changes seen in late AMD and are considered invasive by many patients, which can significantly affect patient compliance. No cure is currently present, and this, along with the widespread prevalence of AMD, is why it is a leading cause of irreversible blindness [36]. MPOD emerges as a potential guardian against the onset and progression of AMD. Macular pigments, which encompass L/Z, exhibit powerful antioxidant properties that counteract the detrimental effects of oxidative stress and inflammation in the retina [6,38]. Lutein and zeaxanthin primarily work by scavenging singlet oxygen and free radicals and mitigating cellular damage in the retina. MPOD contributes to retinal health and reduces the risk of AMD development [6]. Higher MPOD levels are correlated with a decreased risk of both early- and late-stage AMD, highlighting the potential of these pigments in preserving visual function [38,39].

The significance of MPOD transcends its protective role, extending to its potential as a biomarker for AMD risk assessment and progression. Lower MPOD levels have been hypothesized to correlate with an increased likelihood of late AMD development, serving as an early indicator of susceptibility to the disease. Supplementation with lutein/zeaxanthin has been shown to increase MPOD and lower the progression of patients with wet AMD to the late stages of the disease [40]. It is important to note that people diagnosed with AMD have consistently been found to have lower MPOD [41,42,43]. A study by Obana et al. (2008) in a Japanese population reported that macular carotenoids decreased even in older healthy individuals; however, while initial studies are promising, they do not conclusively establish that increasing MPOD reduces the incidence of AMD [44]. Bone et al. examined donor eyes of individuals with and without AMD, finding lower concentrations of L/Z in individuals with AMD [45]. Monitoring MPOD over time may provide valuable insights into disease progression by aiding in identifying individuals who may be at higher risk of transitioning to advanced stages of AMD. Tsika et al. showed a significantly higher MPOD in the fellow eyes of patients with wet AMD, with no difference in the fellow eyes of patients with dry AMD [43]. Integrating MPOD measurements into routine clinical assessments can enhance AMD risk stratification, enabling proactive interventions and personalized management strategies. Table 3 below summarizes the findings of the randomized control trial and observational cross-sectional studies that have examined the relationship between MPOD and AMD, supporting the idea of using MPOD as a clinical biomarker of progression in AMD.

### 3.3. Glaucoma

Glaucoma is the world’s leading cause of irreversible blindness. Glaucoma is characterized by progressive degeneration of the optic nerve head, permanent damage to the retinal nerve fiber layer, and loss of retinal ganglion cells [57]. It results in vision loss that begins peripherally and moves centrally through the course of the disease [57]. In addition to elevated IOP, retinal ischemia, oxidative stress, and damage from ischemia-reperfusion have been proposed as major factors causing retinal ganglion cell death [58]. It has been well established that increased macular pigment density aids in stopping the progression of AMD through potentially anti-oxidative effects [6]. This raises questions regarding the role of the macular pigment in mitigating the oxidative damage seen in glaucoma.

One study injected lutein into a transient ischemia model of high IOP in rats [59]. Rats injected with lutein showed significantly decreased levels of oxidative markers and decreased ischemia-induced retinal cell death compared to controls [59,60]. Research has demonstrated that administering lutein through intravitreal injections to rats suffering from ischemia–reperfusion injuries led to a significant reduction in oxidative markers, an increase in anti-oxidative markers, and a significant decrease in retinal ganglion cell death [61,62]. Müller cells fulfill a dual role by offering homeostatic and metabolic support to retinal neurons while also serving as key mediators of inflammation within the retina [63,64]. A Cross-sectional analysis assessed the MPOD of patients with glaucomatous eyes and found that MPOD was lower in eyes that had a thinner ganglion cell complex, a thinner retinal nerve fiber layer, and an increased cup-to-disc ratio, ultimately indicating a possible correlation between MPOD and glaucoma severity [65]. Further studies are needed to understand this relation better. Studies have primarily focused on assessing the impact of MPOD and L/Z concentration in animal models, yielding promising results [59,60,61,62]. A recent review paper has suggested that carotenoid vitamin therapy provides synergic neuroprotective benefits and has the capacity to serve as adjunctive therapy in the management of glaucoma [66]. However, further research involving human subjects is essential to understand the mechanism and explore the potential anti-oxidative effects, particularly in the context of a high-oxidation disease like glaucoma. Table 4 below summarizes the findings of the randomized control trial and observational cross-sectional studies that have examined the relationship between MPOD and glaucoma, supporting the idea of using MPOD as a clinical biomarker of progression in glaucoma.

### 3.4. Systemic Disease

The assessment of MPOD could be used to gauge the risk of developing ocular and/or systemic disease. At the same time, maintenance or enhancement of MPOD could prevent the onset or advancement of associated co-morbidities. The theorized pathogenic mechanisms and metabolic co-morbidities to explain the lower MP levels reported in diabetes include a process of increased oxidative stress, inflammation, hyperglycemia, insulin resistance or deficiency, obesity, dyslipidemia, and vascular dysfunction/neovascularization [78,79]. In addition to possibly depleting potent antioxidants, such as macular carotenoids lutein, zeaxanthin, and meso-zeaxanthin that are pertinent for retinal protection, these factors may have related and/or independent relationships with MP that warrant further study [78,79]. A significant inverse correlation between MPOD and HbA1C was found, and decreased MPOD is evident in type II diabetes with or without retinopathy [80]. Currently, there is robust evidence and early clinical trials supporting the use of carotenoid vitamin supplementation in diabetics with and without retinopathy. A trial on mice demonstrated promising effects on the prevention of diabetic retinopathy with MPOD-bolstering supplements; by reducing apoptosis of retinal ganglion cells, astaxanthin may prevent oxidative stress from causing retinal neurodegeneration [81]. Table 5 below summarizes the findings of the randomized control trial and observational cross-sectional studies that have examined the relationship between MPOD and diabetes, supporting the idea of using MPOD as a clinical biomarker of progression in diabetes and diabetic retinopathy.

#### Diabetic Retinopathy

Retinopathy is a common ocular complication of uncontrolled type I and type II diabetes. People recently diagnosed with diabetes have been shown to have significantly lower L/Z plasma concentrations [92]. Studies have commonly examined the effects of carotenoids on the development of diabetes, but there is limited research examining the effects of carotenoids on diabetic retinopathy. An animal study conducted by Kowluru et al. examined the effects of zeaxanthin supplementation on the retina in diabetic rats, and the results showed significant inhibition of diabetes-induced retinal oxidative damage [93]. A study conducted by Chous et al. showed that compared to a placebo, subjects taking a xanthophyll multicomponent nutritional supplement demonstrated a 27% increase in MPOD after six months, which correlated with an improvement in visual function, serum lipids, and a decrease in peripheral neuropathy [88]. As discussed earlier, increased MPOD has been shown to mitigate oxidative processes in the retina, and studies have shown that type II diabetes patients have lower levels of MPOD [85]. MPOD levels have also been correlated with HbA1C levels [80]. Evidence regarding MPOD’s effect on the treatment and development of diabetic retinopathy is limited. Early studies and animal models suggest a potential protective role of MPOD in the development of diabetic retinopathy [80,88,93]. 

### 3.5. Visual Performance

MPOD’s influence on visual performance resonates with its role as a natural optical filter. Higher pigment density corresponds to enhanced light absorption, which aids in optimizing contrast sensitivity—the ability to discern subtle differences in light and dark areas [94,95,96,97]. Studies have demonstrated a positive correlation between MPOD and contrast sensitivity, particularly in conditions of low light and reduced contrast [80,88,93]. This correlation underscores the potential of MPOD to fine-tune visual acuity, translating to improved day-to-day activities such as reading, driving, and recognizing facial expressions.

Glare, often experienced as visual discomfort caused by intense light sources, can significantly impede visual function. MPOD’s protective role against glare becomes evident as the pigments selectively filter high-energy blue light, thus reducing glare’s impact on visual perception [94,98]. 

Moreover, MPOD aids in hastening recovery from photostress—the temporary blinding effect experienced after exposure to bright light [94,98]. The pigments’ capacity to absorb excess light and dissipate its energy contributes to quicker recovery times, enhancing visual comfort in challenging lighting conditions [94,98].

Increasing MPOD has been linked to a noteworthy enhancement in best corrected visual acuity (BCVA), as indicated by a study by Loughman et al., revealing a significant positive association (r = 0.237) between MPOD levels and BCVA [96]. This finding suggests that interventions aimed at increasing MPOD levels could be promising in enhancing visual acuity, offering a potential avenue for improving the eyesight of individuals with less-than-optimal BCVA [35].

MPOD’s beneficial effects on disability glare, photostress recovery time, and contrast sensitivity are seen across diverse demographics, including certain professional athletes and individuals with low vision. A one-year randomized, double-blind placebo-controlled trial in truck drivers showed that 20 mg of daily lutein supplementation resulted in increased MPOD and contrast sensitivity and decreased disability glare [99]. This underlines the potential commercial uses of increasing and measuring MPOD. There is evidence to support that MPOD can potentially be used as a clinical biomarker for ocular health in the setting of increased screen time and associated short-wavelength light exposure [100]. One study examined the effects of carotenoid supplementation versus a placebo and found that carotenoid supplementation increased MPOD, which was also associated with improvements in headache frequency, eye strain, eye fatigue, and all measured visual performance variables [97]. Richer et al. conducted an RCT to assess the impact of increasing MPOD on night vision in elderly drivers [101]. They found that over a six-month period, participants who took a 14 mg Z + 7 mg L supplement experienced significant improvements in MPOD, glare recovery, contrast sensitivity, and preferred luminance, suggesting that carotenoid supplementation can enhance visual functions important for night driving [101]. In the realm of sports, where visual acuity and contrast sensitivity are paramount, higher MPOD levels could confer a competitive advantage. Improved contrast sensitivity could enhance an athlete’s ability to discern critical visual cues, thereby refining their performance. Conversely, individuals with low vision may experience perceptual deficits due to compromised contrast sensitivity and glare discomfort. There is no consensus on whether high MPOD levels improve vision. See Table 6 below for a summary.

### 3.6. MPOD and Cognitive Function

It has been well established that a higher level of serum and brain carotenoids is associated with improved cognitive function [114,115,116,117]. It is important to note that the carotenoids present in the macular pigment are also widely present in the brain [118]. One study investigated the relationship between MPOD and cognitive function in 4453 adults aged >50 years and found that lower MPOD was associated with poorer performance on the Mini Mental State Examination and on the Montreal Cognitive Assessment [116]. Lower MPOD was also associated with poorer prospective memory and slower reaction times [116]. As seen in Table 7, MPOD and cognitive function have been positively correlated across various mental processes in various age groups, strongly supporting the use of MPOD as a clinical biomarker for cognitive function.

## 4. Discussion

MPOD assessment, once confined to research settings, is finding its stride as a pivotal component of routine eye examinations. Including MPOD measurement within these examinations provides clinicians with a comprehensive snapshot of a patient’s macular health. This insight extends beyond mere pigment quantification, unveiling potential susceptibility to AMD and other ocular conditions. By integrating MPOD assessment into the standard ocular assessment paradigm, clinicians can gain a deeper understanding of an individual’s visual health trajectory, enabling proactive interventions and tailored recommendations.

The potential of MPOD as a predictive marker revolutionizes the landscape of treatment planning. As our understanding of the relationship between pigment density and ocular health deepens, MPOD emerges as a prognostic tool that guides personalized interventions. For instance, individuals with lower MPOD values may benefit from proactive strategies aimed at enhancing pigment density to mitigate AMD risk. Furthermore, MPOD assessment can aid in identifying individuals likely to respond favorably to specific treatments, optimizing therapeutic outcomes, and minimizing potential side effects.

The longitudinal monitoring of disease progression and treatment efficacy is essential in ocular health management. MPOD’s potential in this realm is significant, offering insights into the evolution of macular health over time. By tracking changes in MPOD, clinicians can gauge dietary inadequacies and disease progression in conditions such as AMD and assess the impact of interventions on pigment density. This enables timely adjustments to treatment and management plans, ensuring that patients receive the most effective care. Additionally, MPOD measurements provide an objective parameter for assessing treatment efficacy, supplementing traditional subjective measures of visual function.

Current clinically available technologies for measuring macular pigment optical density (MPOD) lack the ability to estimate lutein and zeaxanthin optical densities, limiting personalized carotenoid supplementation therapies. The introduction of new biomarkers like lutein and zeaxanthin optical densities through technologies such as the Macular Pigment Reflectometer (MPR) could revolutionize precision medicine by providing repeatable MPOD and carotenoid optical density measurements [12]. Unlike heterochromatic flicker photometers (HFPs), the MPR objectively measures MPOD and individual lutein and zeaxanthin components, offering a faster and more precise method that addresses the limitations of current technologies. The MPR utilizes controlled light beams and internal spectrometers to quantify lutein and zeaxanthin optical densities, providing reliable measurements for personalized supplementation strategies [13]. The current market and consumers are not ready for prophylactic personalized vitamin and nutritional therapies, nor do we have clinically available devices that can objectively measure MPOD and its individual components. The cost is the major prohibiting factor in the implementation of such strategies. The current conditions further emphasize the importance, dominance, and need for HFP devices in the measurement of MPOD. 

Integrating MPOD measurement into clinical applications is not merely an addition to the diagnostic toolkit; it is a paradigm shift that empowers clinicians to provide personalized, proactive, and precise ocular care. The ability to predict risk, tailor treatments, and monitor changes in pigment density imbues ocular health management with unprecedented depth. By harnessing MPOD’s potential, clinicians are poised to elevate the standard of care, ensuring that patients receive interventions that are not only evidence-based but also finely tuned to their individual ocular profiles. 

Advances in technology are poised to revolutionize how MPOD is measured and interpreted. Emerging techniques, such as adaptive optics imaging and multi-wavelength Fundus Autofluorescence, offer enhanced spatial resolution and the ability to quantify pigment distribution across the macula with unprecedented detail. These technologies enable researchers to unravel nuances in pigment density and distribution, potentially linking specific pigment patterns to ocular health outcomes. As these methods become more affordable and accessible, the precision and granularity of MPOD assessment are set to soar, enhancing our understanding of its role in visual health.

The trajectory of ocular and systemic health is a marathon rather than a sprint, necessitating long-term studies to unravel the intricacies of MPOD’s relationship with well-being. Longitudinal investigations are key to deciphering the dynamic interplay between MPOD and age-related ocular diseases, tracking changes in pigment density as individuals age and potentially developing early predictive markers for disease onset. These studies also illuminate the temporal dynamics of MPOD alterations due to lifestyle changes, ethnicity [132], interventions, and genetic predispositions. As we venture into the future, long-term studies will anchor our understanding of MPOD’s enduring influence on ocular health.

## Figures and Tables

**Table 1:** L/Z quantities in commonly consumed food.

Food	Trans-Lutein (µg per 100 g)	Trans-Zeaxanthin (µg per 100 g)	L/Z Ratio
Asparagus, cooked	991	0	-
Broccoli, cooked	772	0	-
Cucumber	361	0	-
Spinach, cooked	12,640	0	-
Spinach, raw	6603	0	-
Tomato, raw	32	0	-
Lettuce, romaine	3824	0	-
Lettuce, iceberg	171	12	14.3
Green beans, cooked from frozen	306	0	-
Kale, cooked	8884	0	-
Pepper, orange	208	1665	0.1
Pepper, green	173	0	-
Bread, white	15	0	-
Egg (yolk + white), cooked	237	216	1.1
Egg yolk, cooked	645	587	1.1
Pistachio, shelled	1405	0	-
Grapes, green	53	6	8.8
Cilantro	7703	0	-
Lima beans, cooked	155	0	-
Olive, green	79	0	-
Parsley, raw	4326	0	-
Squash, yellow, cooked	150	0	-
Zucchini, cooked with skin	1355	0	-

Abbreviations: L/Z = lutein/zeaxanthin; μg = microgram. Data obtained from [11].

**Table 2:** Advantages and disadvantages of each MPOD/carotenoid measurement technique.

Method	Advantages	Disadvantages
Heterochromatic flicker photometry (HFP) [16,17]	- Non-invasive and subjective - Widely used and validated	- Subjectivity can lead to variability - Requires significant training
Fundus Reflectometry (FR) [18,19]	- Objective measurement - High sensitivity for detecting small changes	- Complexity and cost of equipment - Accuracy can be compromised by ocular media opacities
Fundus Autofluorescence (FAF) [21,23]	- Non-invasive and quick - Provides additional diagnostic information on retinal health	- Accuracy affected by cataracts - Pathological retinal changes can skew results
Resonance Raman Spectroscopy (RRS) [24,25]	- High specificity for carotenoids - Provides precise quantitative data	- Requires expensive, specialized equipment - Sensitive to patient movement
Veggie Meter [30,31] (**Cannot measure MPOD but has weak correlation to MPOD**)	- Non-invasive and quick - Portable and convenient for screenings - Provides insight into patient diet	- Skin carotenoid scores can be impacted by individual characteristics including age, sex, BMI, smoking status, supplement use, and diagnosed chronic diseases - Highly sensitive to excess heat and light, making it recommended to record environmental conditions - Skin heterogeneity can cause score variability
High-Performance Liquid Chromatography (HPLC) serum carotenoids [34,35] (**Cannot measure MPOD**)	- Precise quantification, especially when analyzing materials for a wide range of organic compounds - Adequately sensitive, repeatable, and suitable for the large-scale analysis of compounds in biological fluids	- Requires longer analysis times - Normal-phase HPLC is not suitable for carotenoid separation due to poor separation of non-polar carotenoids - Temperature-sensitive - Time-consuming - Expensive - Influenced by recent intake of carotenoids and absorption of the tissue

**Table 3:** Randomized control trial studies examining the relationship between MPOD and AMD.

Author (Year)	Study Design	Inclusion Criteria	Sample Size	Interventions	Duration	Relation between MPOD and AMD	MPOD Technique
Beatty (2013) [46]	RCT	Adults ≥55 years with early- or late-stage AMD.	433	Group 1: L and Z, Vitamin C, Vitamin E, Copper, Zinc. Group 2: Placebo.	Minimum 12 months, up to 36 months	Supplementation with L, Z, and antioxidants showed functional and morphologic benefits in early AMD. MPOD increased in the active group and decreased in the placebo group.	RS
LUTEGA study (2013) [47]	RCT	Adults 60–80 years with non-exudative AMD.	172	Group 1: L, Z, Omega-3, antioxidants. Group 2: Placebo	12 months	Supplementation resulted in a considerable increase in MPOD and improvement/stabilization in BCVA. There was no difference in MPOD accumulation between dosages.	FA
CLEAR study (2013) [48]	RCT	Adults 50–80 years with early AMD.	72	Group 1: L (10 mg) Group 2: Placebo	12 months	Lutein supplementation increased MPOD and may have a mild beneficial effect on visual acuity. No change in MPOD was found in the placebo group.	HFP
LAST study (2004) [49]	RCT	Adults 55–80 years with atrophic AMD.	90	Group 1: L (10 mg) Group 2: L (10 mg) with antioxidants Group 3: Placebo	12 months	Lutein alone or with antioxidants improved MPOD, glare recovery, and contrast sensitivity. No significant change was found in the placebo group.	HFP
LUNA study (2007) [50]	RCT	Adults ≥ 55 years with or without AMD.	120	Group 1: L (6 mg) Group 2: Placebo	6 months	Lutein supplementation increased MPOD and improved visual function. No change was found in the placebo group.	FA
ZVF study (2011) [51]	RCT	Early and moderate AMD retinopathy, symptoms of visual deficits.	60	Group 1: Z (8 mg) Group 2: Z (8 mg) + L (9 mg), Group 3: Placebo	12 months	MPOD increased in the intervention groups compared to the placebo group.	HFP
Weigert (2011) [52]	RCT	Adults 50–90 years with AREDS stages 2, 3, and 4.	126	Group 1: L (20 mg for first 3 months, then 10 mg) Group 2: Placebo	6 months	Lutein significantly increased MPOD by 27.9%. No significant effect on macular function or visual acuity was observed.	HFP
Sabour-Pickett (2014) [53]	RCT	Adults 50–79 years with early AMD.	52	Group 1: L (20 mg) and Z (2 mg) Group 2: MZ (10 mg), L (10 mg), Z (2 mg) Group 3: MZ (17 mg), L (3 mg), Z (2 mg)	12 months	A statistically significant increase in MPOD was observed in Group 2 and Group 3. Improvements in letter contrast sensitivity were seen in all groups, with the best results in Group 3.	HFP
Huang (2015) [54]	RCT	Adults 50–79 years with early AMD.	112	Group 1: L (10 mg) Group 2: L (20 mg) Group 3: L (10 mg) and Z (10 mg)	2 years	All active treatment groups showed a significant increase in MPOD. The 20 mg lutein group was the most effective at increasing MPOD and contrast sensitivity at 3 cycles/degree for the first 48 weeks.	FA
Davey (2020) [55]	RCT	Adults 50–79 years with retinal drusen.	56	Group 1: Lumega-Z softgel Group 2: PreserVision AREDS2 softgel	6 months	Both groups demonstrated statistically significant improvements in contrast sensitivity function (CSF) in both eyes at six months.	HFP
Ma (2012) [56]	RCT	Ages 50–79, early AMD.	108	Group 1: L (10 mg) Group 2: L (20 mg) Group 3: L (10 mg) plus Z (10 mg)	48 weeks	There was a significant increase in MPOD in the high-dose lutein and lutein-plus-zeaxanthin groups, with improvements in contrast sensitivity at certain spatial frequencies.	FA

Abbreviations: RCT = randomized control trial; AMD = age-related macular degeneration; L = lutein; Z = zeaxanthin; MZ = meso-zeaxanthin; BCVA = best corrected visual acuity; RS = Raman Spectroscopy; HFP = heterochromatic flicker photometry; FA = Fundus Autofluorescence.

**Table 4:** Cross-sectional and randomized control trial studies examining the relationship between MPOD and glaucoma.

Author (Year)	Study Design	Inclusion Criteria	Sample Size	Intervention(s)	Duration	Relation between MPOD and Glaucoma	MPOD Technique
Fikret (2021) [67]	CS	Age not mentioned. Patients with POAG, PEX, and controls.	79	None	N/A	Higher MPOD values in patients with PEX glaucoma; no significant differences in POAG compared to controls. There was no correlation between MPOD values and RNFL or GCL.	FR
Bruns (2020) [68]	CS	Adults 34–87 years. Patients with POAG and controls.	86	None	N/A	No significant difference in MPOD values between POAG patients and controls.	DWA
Loughman (2021) [69]	RCT	Adults > 18 years. Patients with POAG and controls.	62	Group 1: L (10 mg) + Z (2 mg) + MZ (10 mg). Group 2: Placebo.	18 months	Supplementation led to a significant increase in MPOD volume. No clinically meaningful changes were noted in glaucoma parameters.	DWA
Siah (2015) [65]	CS	Adults 36–84 years. Patients with POAG and controls.	88	None	N/A	Lower MPOD was observed in glaucomatous eyes compared to controls. Worse glaucomatous parameters were observed in patients with lower MPOD.	HFP
Ji (2016) [70]	CS	Adults 20–76 years. Patients with POAG and controls.	82	None	N/A	MPOD was significantly lower in POAG patients compared to controls and correlated positively with GCC thickness.	FR
Arnould (2022) [71]	CS	Adults >75 years. Patients with POAG and controls.	1153	None	N/A	No significant differences in MPOD were found between the POAG group and the control group.	DWA
Daga (2018) [72]	CS	Adults 20–76 years. Patients with POAG and controls.	107	None	N/A	No significant association was found between MPOD volume and glaucoma status.	DWA
Lawler (2023) [73]	CS	Adults 55–81 years. Patients with POAG and controls.	379	None	N/A	MPOD was positively associated with GCC and GCL, among POAG and controls.	HFP
Igras (2013) [74]	CS	Adults 58–80 years. Patients with POAG and controls.	40	None	N/A	MPOD was significantly lower in POAG patients compared to controls.	HFP
Siah (2018) [75]	CS	Adults 36–84 years. Patients with POAG and controls.	88	None	N/A	MPOD was associated with improved glare-affected visual function and less central visual field loss in POAG patients.	HFP
Liu 2024 [76]	CS	Adults 69–98 years. Patients with POAG and controls.	26	None	N/A	Glaucomatous eyes had 25% lower MPOD compared to non-glaucomatous eyes.	HFP
Eraslan (2023) [77]	CS	Adults >55 years. Patients with POAG currently receiving topical medication and controls.	52	None	N/A	MPOD levels were higher in POAG patients compared to controls, suggesting a possible protective effect of topical therapies.	FR

Abbreviations: RCT = randomized control trial; CS = cross-sectional; POAG = primary open-angle glaucoma; L = lutein; Z = zeaxanthin; MZ = meso-zeaxanthin; HFP = heterochromatic flicker photometry; FR = Fundus Reflectance; DWA = dual-wavelength autofluorescence; RNFL = retinal nerve fiber layer; GCL = ganglion cell layer thickness; PEX = pseudoexfoliative; GCC = ganglion cell complex.

**Table 5:** Cross-sectional and randomized control trial studies examining the relationship between MPOD and diabetes.

Author (Year)	Study Design	Inclusion Criteria	Sample Size	Intervention(s)	Duration	Relation between MPOD and DR	MPOD Technique
Lima (2010) [82]	CS	Adults 56–63; BCVA ≤20/40.	43	None	N/A	MPOD was lower in diabetic patients, with a significant inverse correlation with HbA1C levels.	DWA
Scanlon (2019) [83]	CS	Adults 50+; BCVA ≤20/40.	2782	None	N/A	MPOD was found to be lower in individuals with T2D compared to healthy controls.	HFP
Bikbov (2015) [84]	CS	Adults 55–71; BCVA ≤20/40.	52	None	N/A	Significant reduction in MPOD in patients with diabetic macular edema compared to controls.	FR
Scanlon (2015) [85]	CS	Adults 36–73; BCVA ≤20/25.	150	None	N/A	MPOD was significantly lower in T2D compared to T1D and controls. The diabetes control was not associated with MPOD.	HFP
She (2016) [86]	CS	Adults over 55–71; BCVA ≤20/25.	401	None	N/A	No significant difference in MPOD levels among groups with or without early-stage non-proliferative DR.	HFP
Bikbov (2015) [87]	CS	Adults 54–69; BCVA ≤20/25.	31	None	N/A	Significant reduction in MPOD in DME patients and strong inverse correlation between retinal thickness and MPOD.	FR
Chous (2016) [88]	RCT	Adults 43–69; BCVA ≥20/30; no or mild-to-moderate DR.	67	Group 1: Carotenoid supplement Group 2: Placebo	6 months	Supplemented group showed significant improvements in visual functions which correlated with increased MPOD compared to the placebo.	HFP
Zagers (2005) [89]	CS	Adults 23–61; BCVA ≤20/32.	14	None	N/A	No significant difference in MPOD density between diabetic patients and healthy controls.	FR
Varghese (2019) [90]	CS	Adults 49–54 years.	150	None	N/A	MPOD was similar across diabetic patients with and without DR, suggesting no significant difference due to DR.	FR
Cennamo (2019) [91]	CS	Adults 31–38 years; T1D and controls.	59	None	N/A	MPOD and vessel density were both significantly lower in diabetic patients compared to controls. There was a moderate correlation between vessel density and MPOD.	FR

Abbreviations: RCT = randomized control trial; CS = cross-sectional; DR = diabetic retinopathy; BCVA = best corrected visual acuity; T1D = type 1 diabetic; T2D = type 2 diabetic; HFP = heterochromatic flicker photometry; DWA = dual-wavelength autofluorescence; FR = Fundus Reflectance.

**Table 6:** Cross-sectional and randomized control trial studies examining the relationship between MPOD and visual function.

Author (Year)	Study Design	Demographic	Sample Size	Interventions	Duration	Relation between MPOD and Visual Function	MPOD Technique
Stringham (2011) [98]	CS	Adults 23–50; BCVA ≤20/25.	26	None	N/A	MPOD was associated with faster photostress recovery, lower disability glare thresholds, and reduced visual discomfort.	HFP
Engles (2007) [102]	CS	Adults 18–40; BCVA ≤20/40.	80	None	N/A	No significant correlation was found between MPOD and measures of visual acuity.	HFP
Tudosescu (2018) [103]	CS	Adults 18–65 years; BCVA ≤20/125.	83	None	N/A	No significant correlation between MPOD and blue-light exposure from computers, iris color, refractive errors, or glare sensibility was found.	HFP
Patryas (2014) [104]	CS	Adults 18–68 years; BCVA ≤20/32.	33	None	N/A	MPOD was weakly associated with rod-mediated recovery, but not with cone-mediated recovery.	HFP
Bovier (2014) [105]	RCT	Adults 18–32 years; BCVA ≤20/60.	92	Group 1: Z—20 mg Group 2: Mixed (Z—26 mg, L—8 mg, Omega-3—190 mg) Group 3: Placebo	4 months	MPOD increased with supplementation and led to significant improvements in visual processing speed and motor reaction time.	HFP
Kvansakul (2006) [95]	RCT	Adults 18–40 years; BCVA ≤20/60.	92	Group 1: L—10 mg Group 2: Z—10 mg Group 3: Combination (L—10 mg, Z—10 mg) Group 4: Placebo	12 months	Supplementation with L or Z increases MPOD and improved contrast acuity thresholds at high mesopic levels, thus enhancing visual performance at low illumination.	HFP
Putnam (2015) [106]	CS	Adults 18–35 years; BCVA ≤20/25.	33	None	N/A	Increased MPOD correlates with reduced glare disability, significantly at higher spatial frequencies.	HFP
Stringham (2008) [107]	RCT	Adults 17–41 years.	40	Group 1: L—10 mg, Z—2 mg Group 2: Placebo	6 months	Supplementation led to increased MPOD, which significantly improved performance in glare disability and photostress recovery tasks.	HFP
Stringham (2017) [97]	RCT	Adults 18–25 years.	59	Group 1: L—6 mg and Z—6 mg Group 2: L—12 mg and Z—12 mg Group 3: Placebo	12 months	Increases in MPOD led to improved contrast sensitivity.	HFP
Nolan (2016) [108]	RCT	Adults with a mean age of 21.5 years.	105	Group 1: L—10 mg, Z—2 mg, and MZ—10 mg Group 2: Placebo	12 months	MPOD increased with supplementation and was significantly correlated with improvements in contrast sensitivity in the active group compared to the placebo.	DWA
Hammond (2014) [109]	RCT	Adults 20–40 years.	115	Group 1: L—10 mg, Z—2 mg Group 2: Placebo	12 months	Supplementation increased MPOD significantly, improving chromatic contrast and photostress recovery time, but glare disability improvements were not statistically significant.	HFP
Hammond (2013) [94]	CS	Adults 20–40 years.	150	None	N/A	MPOD density significantly correlated with positive outcomes in glare disability, photostress recovery time, and chromatic contrast thresholds.	HFP
Stringham (2016) [110]	RCT	Adults 18–25 years, BCVA ≤20/20.	59	Group 1: L—10 mg + Z—2 mg Group 2: L—20 mg + Z—4 mg Group 3: Placebo	12 months	Supplementation led to significant increases in MPOD, which in turn resulted in improvements in photostress recovery and disability glare.	HFP
Hammond (1998) [111]	CS	Adults 60–84 years; ≤20/32 visual acuity.	37	None	N/A	A higher MPOD was associated with preserved visual sensitivity in older ages.	HFP
Estévez-Santiago (2016) [112]	CS	Adults 20–35 and 45–65 years; BCVA ≤20/20.	108	None	N/A	The contrast threshold was inversely correlated with MPOD, particularly in the older group.	HFP
Nolan (2011) [113]	RCT	Adults 18–41 years; BCVA ≤20/20.	121	Group 1: L—12 mg + Z—1 mg Group 2: Placebo	12 months	A statistically significant increase in MPOD in the active group was not generally associated with improvement in visual performance.	HFP
Loughman (2010) [96]	CS	Adults 18–41 years; BCVA ≤20/20.	142	None	N/A	MPOD was positively associated with BCVA and contrast sensitivity, while photostress recovery and glare sensitivity were unrelated to MPOD.	HFP

Abbreviations: RCT = randomized control trial; CS = cross-sectional; L = lutein; Z = zeaxanthin; MZ = meso-zeaxanthin; BCVA = best corrected visual acuity; HFP = heterochromatic flicker photometry; DWA = dual-wavelength autofluorescence.

**Table 7:** Cross-sectional and randomized control trial studies examining the relationship between MPOD and cognitive function.

Author (Year)	Study Design	Inclusion Criteria	Sample Size	Intervention(s)	Duration	Relation between MPOD and Cognitive Function	MPOD Technique
Khan (2018) [119]	CS	Adults 25–45 years with BMI ≥ 25 kg/m^2^.	114	None	N/A	MPOD positively associated with IQ and fluid intelligence, but not with crystallized intelligence.	HFP
Saint (2018) [120]	CS	Children 7–13 years.	51	None	N/A	MPOD positively associated with reasoning skills and executive mental processes.	HFP
Renzi-Hammond (2017) [121]	RCT	Adults 18–30 years.	51	Group 1: L (10 mg) + MZ (2 mg). Group 2: Placebo	1 year	MPOD positively associated with improvements in spatial memory, reasoning ability, and complex attention tasks.	HFP
Barnett (2018) [122]	CS	Preadolescent children 8–9 years.	56	None	N/A	MPOD positively associated with overall academic achievement, mathematics, and written language.	HFP
Lindbergh (2018) [123]	RCT	Adults 64–86 years.	44	Group 1: L (10 mg) + MZ (2 mg). Group 2: Placebo	1 year	L and Z supplementation increased MPOD and was associated with enhanced signals in prefrontal regions, suggesting a potential mechanism for improved cognitive performance.	HFP
Kelly (2015) [124]	CS	Group 1: Adults 35–74 years with low MPOD. Group 2: Adults 35–74 years with early AMD.	226	None	N/A	MPOD positively associated with phonemic fluency, attention switching, visual and verbal memory, and learning.	HFP and DWA
Power (2018) [116]	RCT	Adults 33–57 years with low MPOD.	91	Group 1: L (10 mg) + MZ (10 mg) + Z (2 mg). Group 2: Placebo	12 months	Supplementation improved MPOD, which was positively associated with episodic memory and overall cognitive function.	DWA
Ajana (2018) [125]	CS	Adults 75–93 years with low MPOD.	184	None	N/A	Higher MPOD was significantly associated with better global cognitive performance, visual memory, and verbal fluency.	DWA
Vishwanathan (2014) [126]	CS	Adults 75–80 years.	108	None	N/A	MPOD levels were significantly positively associated with better global cognition, verbal learning and fluency, recall, processing speed, and perceptual speed.	HFP
Renzi (2014) [127]	CS	Adults 65–83 years with mild cognitive impairment.	53	None	N/A	In unimpaired adults, higher MPOD was associated with better visuospatial and constructional abilities. In mildly impaired adults, higher MPOD was associated with better performance in multiple cognitive domains including memory, language, and attention.	HFP
Feeney (2013) [118]	CS	Adults 50+ years.	4453	None	N/A	Lower MPOD was associated with poorer performance on the MMSE and MoCA, prospective memory, and executive function.	HFP
Stringham (2019) [128]	RCT	Adults 18–25 years.	59	Group 1: MZ (13 mg) Group 2: MZ (27 mg) Group 3: Placebo	6 months	Supplementation improved cognitive performance in composite memory, verbal memory, sustained attention, psychomotor speed, and processing speed.	HFP
Hassevoort (2017) [129]	CS	Children 7–10 years.	40	None	N/A	MPOD was negatively associated with relational memory errors.	HFP
Edwards (2019) [130]	CS	Adults 25–45 years with BMI ≥ 25 kg/m^2^.	101	None	N/A	MPOD was positively associated with improvements attentional resource allocation and information processing speed.	HFP
Mewborn (2018) [131]	CS	Adults 64–77 years.	51	None	N/A	Higher MPOD was positively associated with better neural efficiency in visual–spatial processing.	HFP

Abbreviations: RCT = randomized control trial; AMD = age-related macular degeneration; L = lutein; Z = zeaxanthin; MZ = meso-zeaxanthin; MMSE = Mini Mental State Examination; MoCA = Montreal Cognitive Assessment; HFP = heterochromatic flicker photometry; DWA = dual-wavelength autofluorescence.

## Data Availability

Dataset available on request from the authors.
